# Supplementary material for: Coalescence and directed anisotropic growth of starch granule initials in subdomains of Arabidopsis thaliana chloroplasts
Source: Nat Commun. 2021 Nov 26;12:6944. doi: 10.1038/s41467-021-27151-5 (PMC8626487; doi:10.1038/s41467-021-27151-5)
Supplement: Supplementary file 1 — Supplementary Information [file 41467_2021_27151_MOESM1_ESM.pdf]

Supplementary Materials for

Coalescence and directed anisotropic growth of starch granule initials in subdomains of *Arabidopsis thaliana* chloroplasts

Léo Bürgy, Simona Eicke, Christophe Kopp, Camilla Jenny, Kuan Jen Lu, Stephane Escrig, Anders Meibom, Samuel C. Zeeman
Correspondence to: samuel.zeeman@biol.ethz.ch

This PDF file includes:

Supplementary Figs. 1 to 6

Other Supplementary Materials for this manuscript include the following:

Supplementary Movies 1 to 4

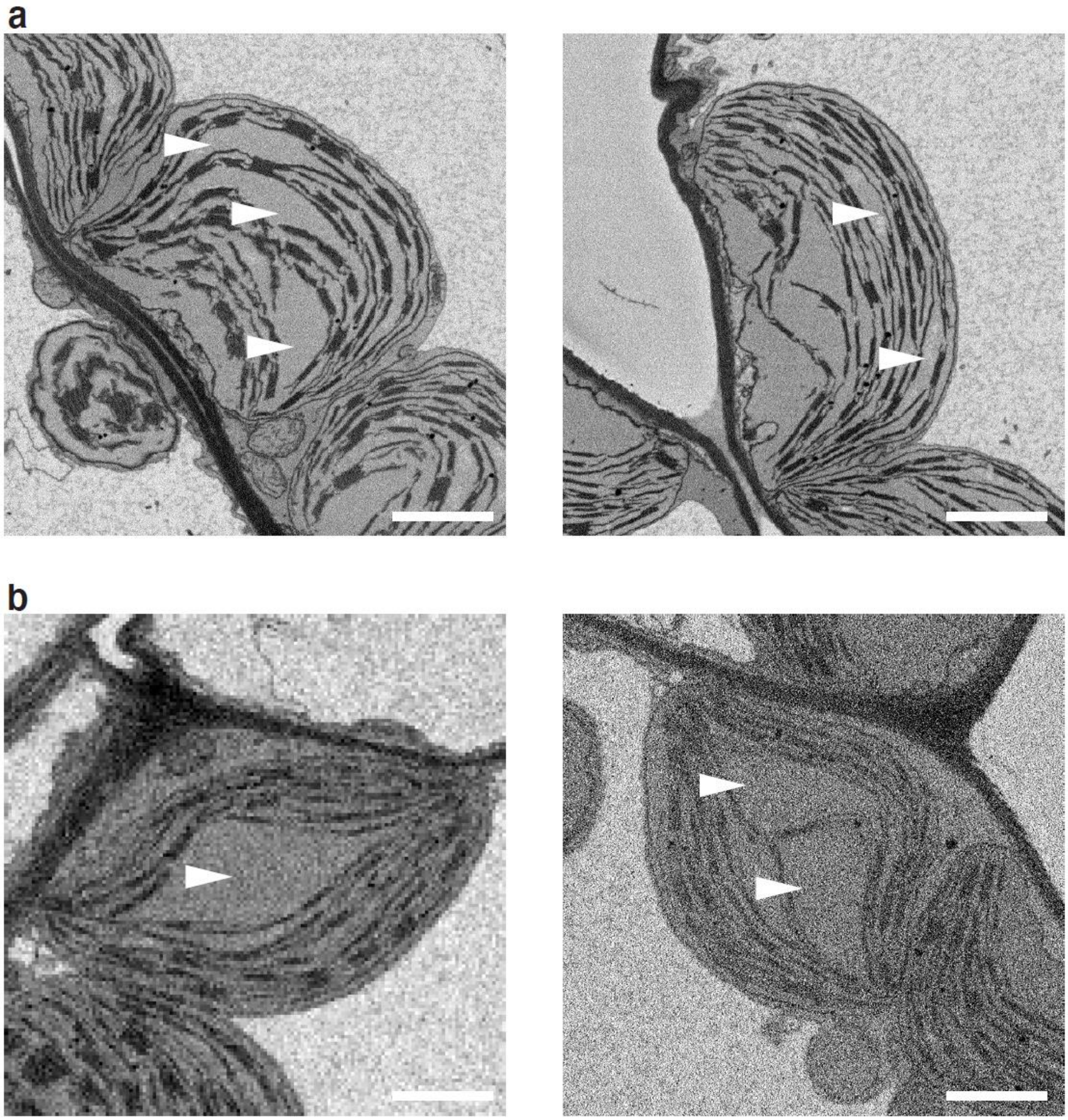

Supplementary Fig. 1.

Additional examples of empty pockets in chloroplasts. Plants were exposed to a 4h-night extension and harvested immediately without further exposure to light, Examples are from two biological replicates (**a** and **b**). The arrowheads point to the putative stromal pockets. Scale bars: 2 µm.

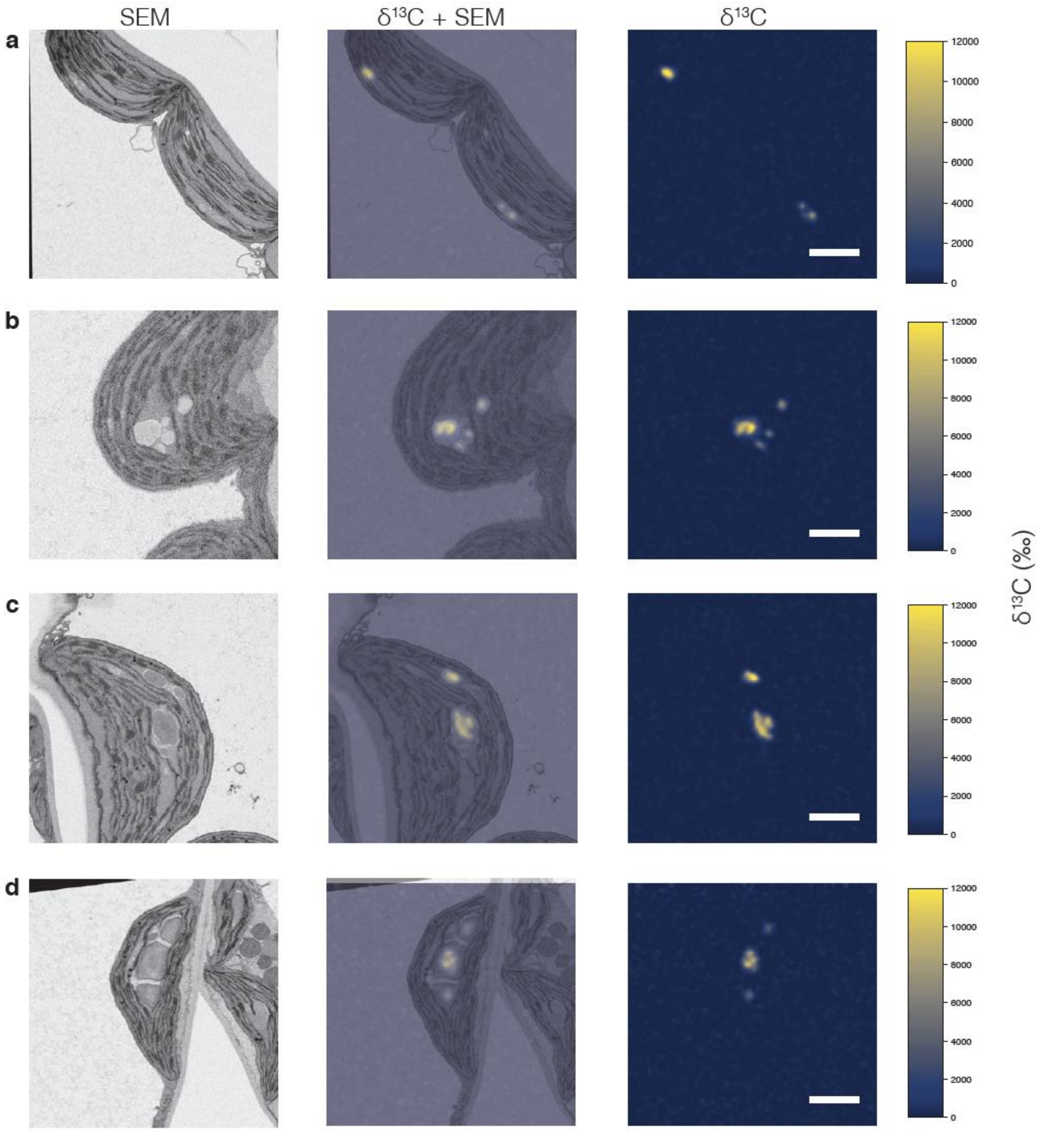

Supplementary Fig. 2.

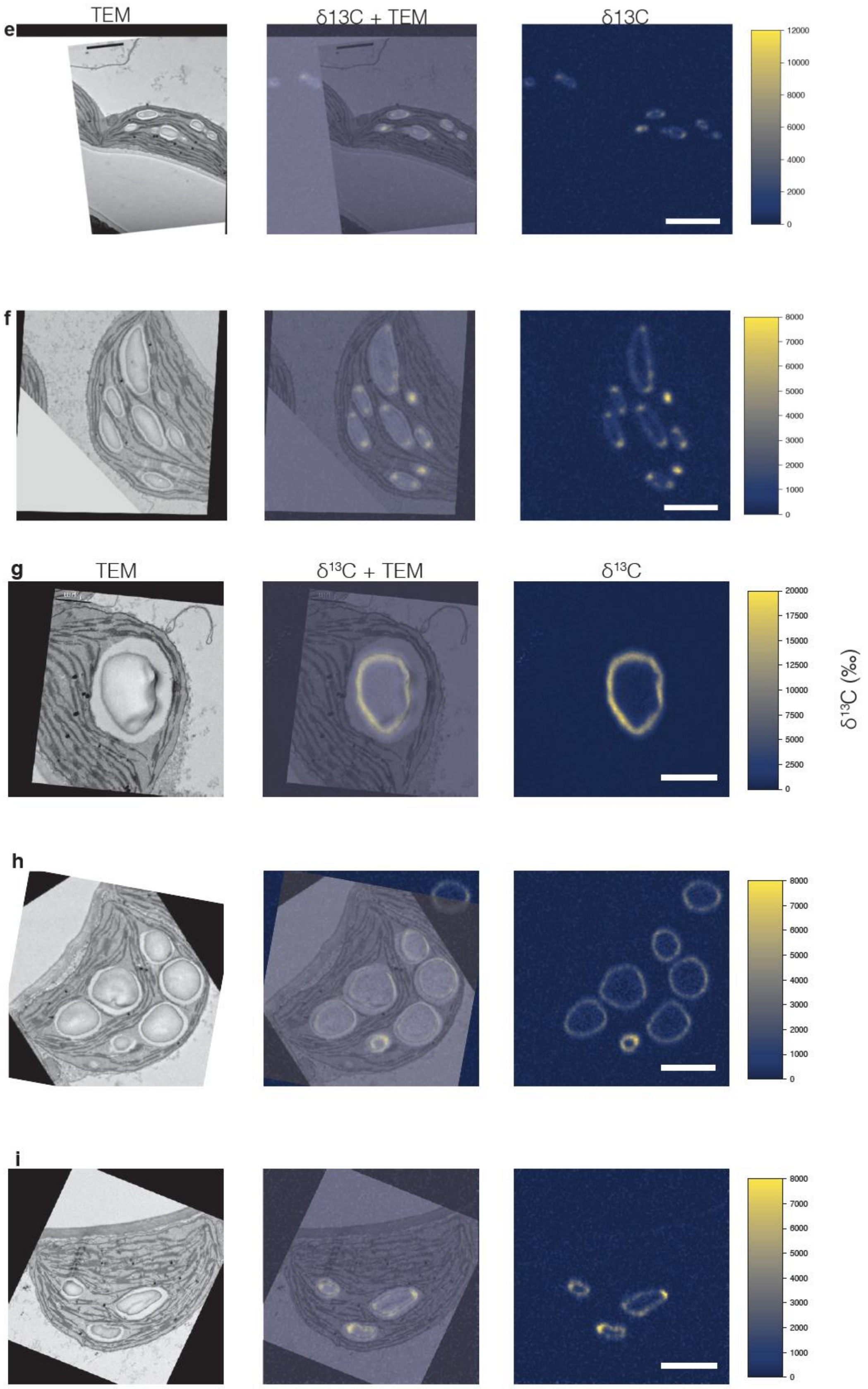

Supplementary Fig. 2 cont.

Supplementary Fig. 2.

Additional examples of starch granule development visualized by $^{13}CO_2$ labelling, EM and NanoSIMS. (**a**–**d**) Additional examples of starch granule coalescence in wild-type chloroplasts, as described in Fig. 3 a–d, respectively. (**e**–**f**) Additional examples of anisotropic granule expansion in wild-type chloroplasts, as described in Fig. 4. (**g**–**i**) Additional examples of aberrant granule initiation and growth in *ss4* mutant chloroplasts (**g**) and the rescue of these phenotypes in the chloroplasts *ss4* plants transformed with *A. tumefaciens* GS (**h**) or GS fused to the N-terminus of SS4 (**i**), as described in Fig. 5. Scale bars: 2 µm.

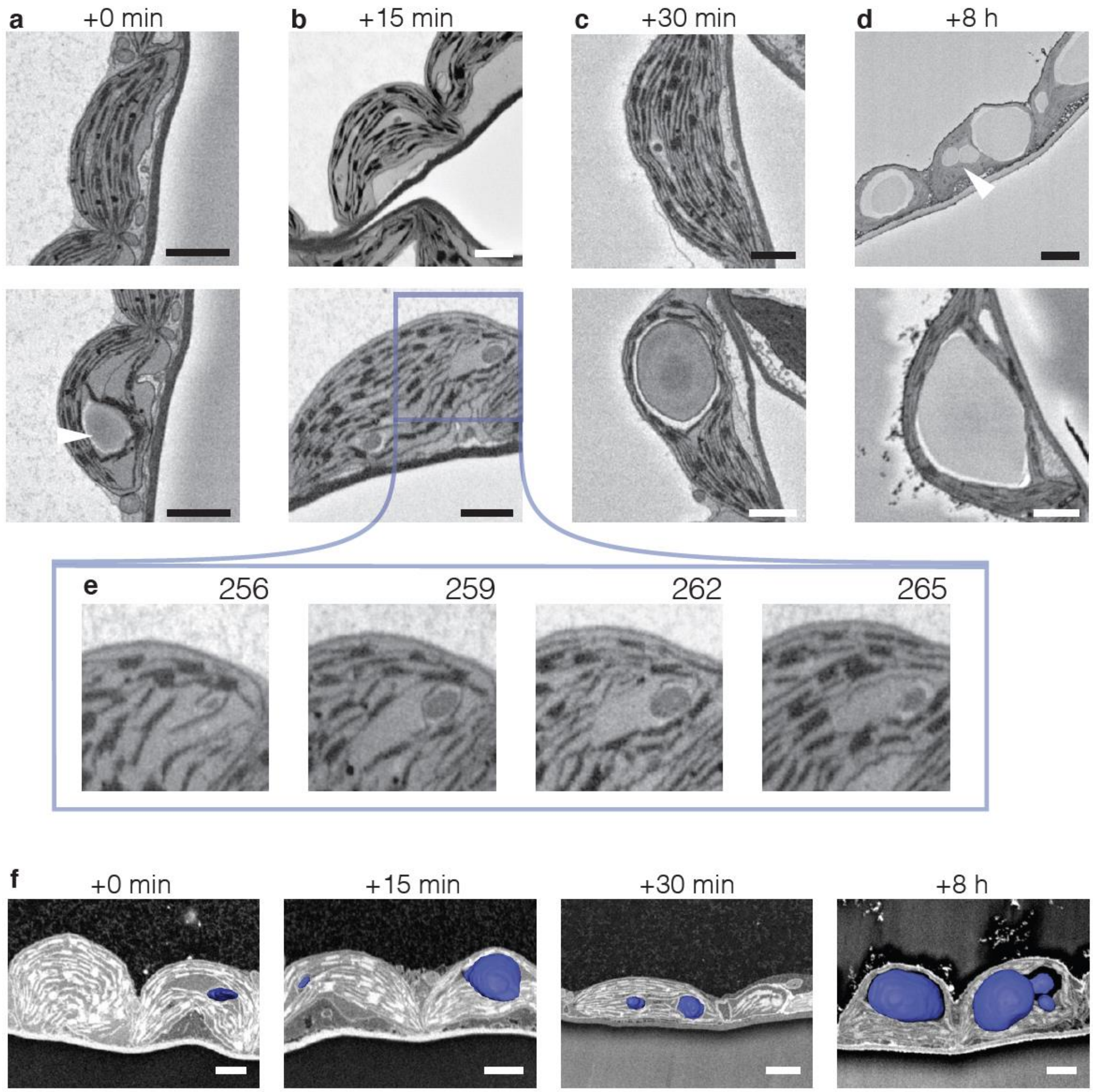

Supplementary Fig. 3.

The *ss4* mutant displays aberrant starch granule initiation. Chloroplast sections from an SBF-SEM stack (with 50-nm Z-resolution, inverted SEM images) sampled from the mesophyll of fully expanded leaves of 35-day old *ss4* mutant plants grown in a 12 h:12 h diel regime. Few, near-spherical starch granules are present between the thylakoid membranes. (**a**–**d**) Representative chloroplast sections from plants exposed to a prolonged night and harvested (**a**) at the end of the 4-h night extension, with a remaining starch granule visible in the lower panel (white arrowhead), (**b**) after 15 min and (**c**) 30 min light, where both large granules and possible newly-initiated granules (white arrowhead) could be observed, and (**d**) after 8 h light, where a mixture of large and small granules were visible. (**e**) Series of images (with steps of 150 nm in the Z-axis) from the sample stack in (b) illustrating a potential newly initiated granule. (**f**) 3D-renderings of representative chloroplasts (non-inverted SEM images) with their starch granules shown in violet. Scale bars: 2 µm.

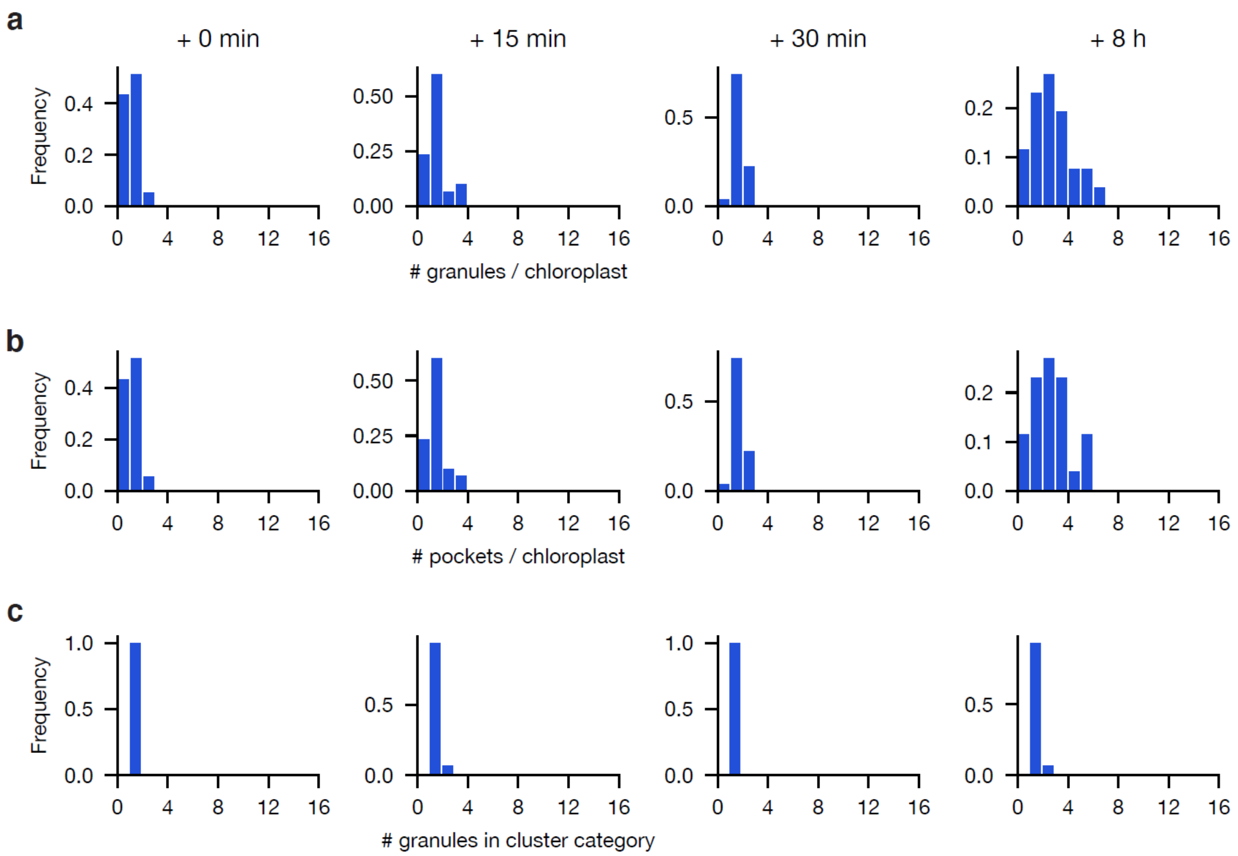

Supplementary Fig. 4.

Determination of the number of starch granules in the *ss4* mutant. Quantitative information was obtained from image stacks as shown in Supplementary Fig. 2. (**a**) The number of granules per *ss4* chloroplast. (**b**) The number of starch-containing pockets per *ss4* chloroplast. (**c**) Number of granules in each cluster category. Data in A–C are expressed as frequency distributions. For each time point, two plants from two independent experiments were examined. In total, 37, 30, 27 and 20 chloroplasts were examined, respectively, for the 0-min time point, i.e., the end of the extended night, and for the 15-min, 30-min and 8-h time points during the day.

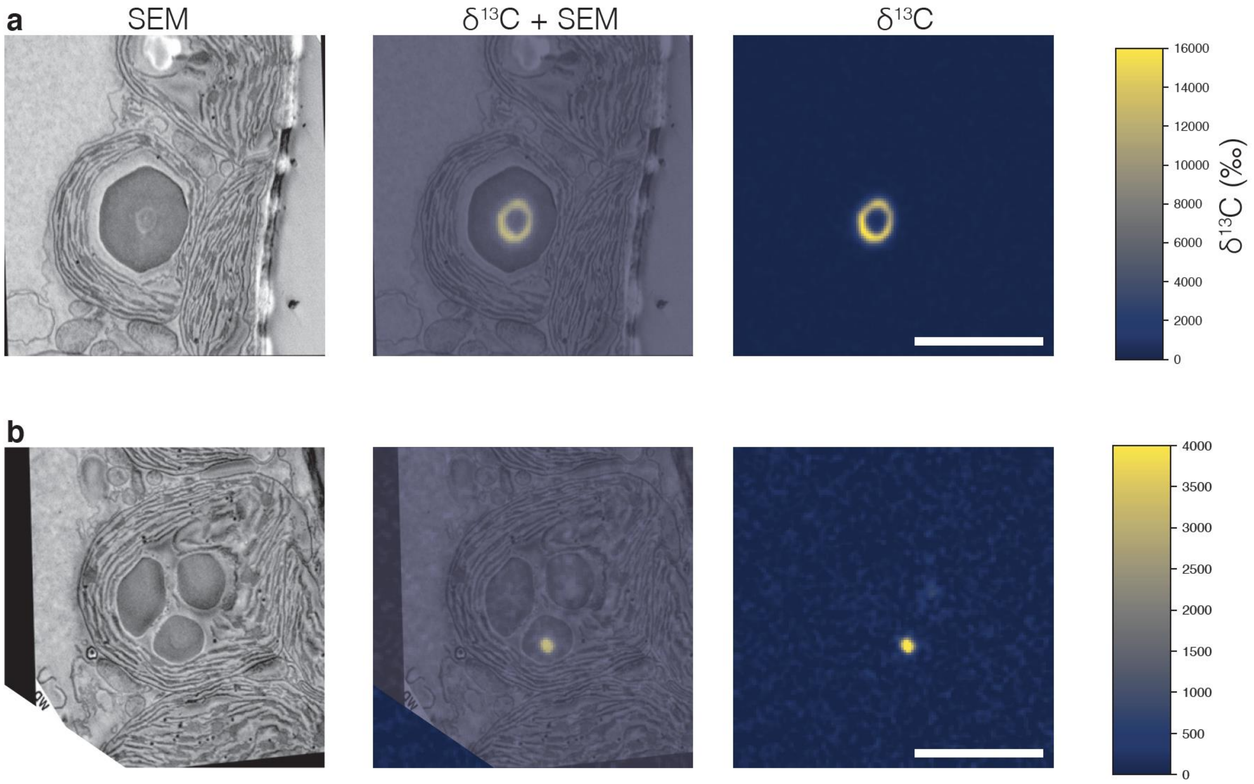

Supplementary Fig. 5.

Re-expansion of existing starch granules and initiation of new granules in the *ss4* mutant. *ss4* mutant plants were subjected to a 4-h night extension and labelled with a pulse of $^{13}CO_2$ for 15 min in the light, then harvested and fixed for EM and NanoSIMS imaging after a chase of 4 h in air in the light. In some cases (**a**), the ^{13}C-enrichment pattern was indicative of peripheral labelling of a pre-existing granule that remained after the extended night (see Supplementary Fig. 2A). In other cases, strong ^{13}C-enrichment was observed at the center of the granule, consistent with the initiation of a new starch granule during the pulse (**b**). Note that since some *ss4* chloroplasts retain starch even after a dark extension, we cannot state with certainty that such granules were newly initiated that day because we do not know the plane of section. If sectioned through their centers, i.e., a great circle spheric section, this would imply new initiation, but if a larger granule was sectioned off-center, i.e., a small circle spheric section, peripheral labelling could be miss-interpreted as a central labelling of a newly initiated granule. However, it is likely that such central labelling would be weak and diffuse. Scale bars: 2 µm.

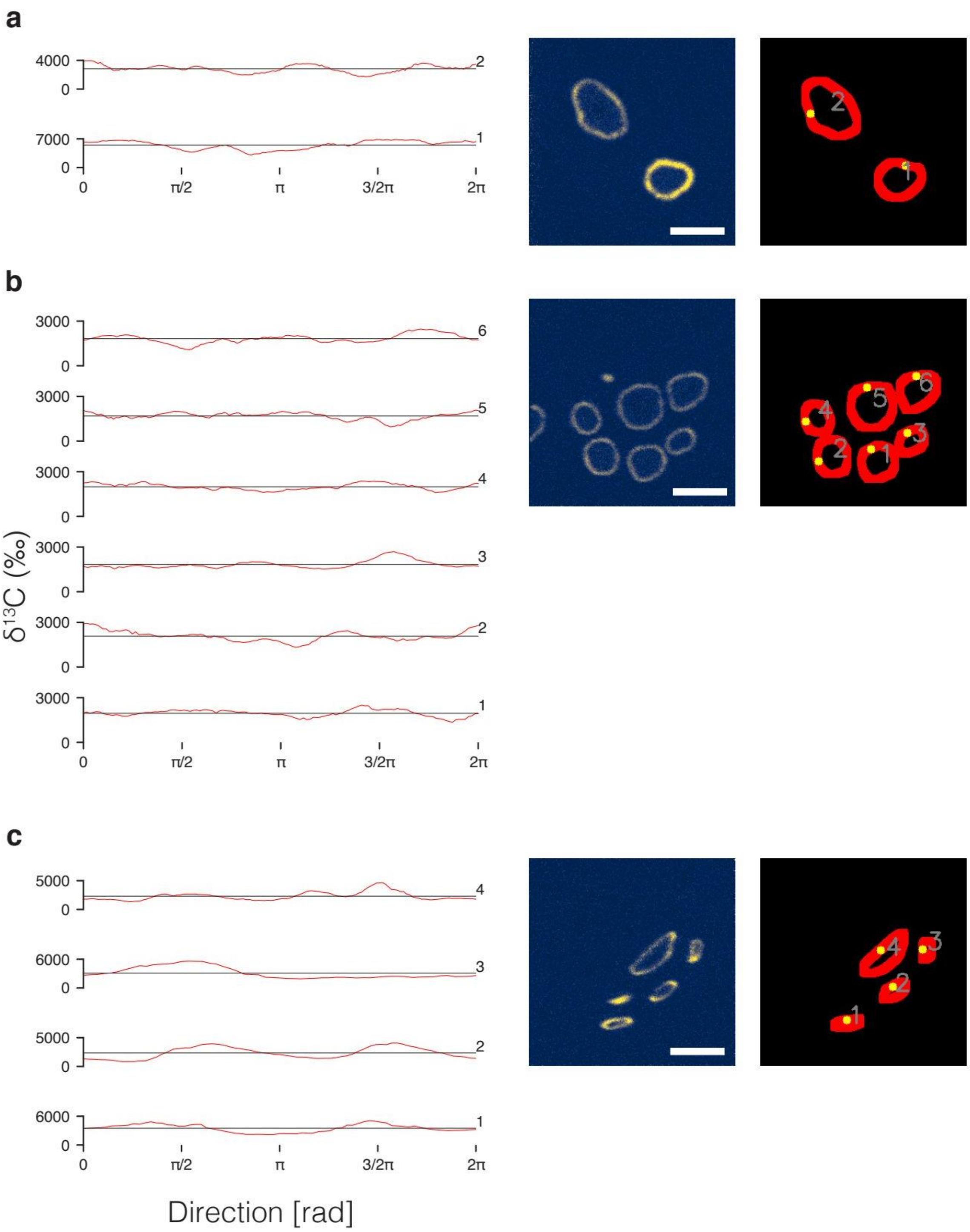

Supplementary Fig. 6. Quantification of ^{13}C-enrichment on the surface of the granule sections depicted in Fig. 5. The probing regions (depicted in red in the inset, right) were defined manually with masks over the δ^{13}C map. Individual circular profiles were extracted anticlockwise from the starting point (yellow). Horizontal lines show the mean enrichment for each profile. (**a**) Chloroplasts from the *ss4* mutant. (**b**) Chloroplasts from the *ss4* mutant expressing a self-glycosylating glycogen synthase (GS) from *A. tumefaciens*. (**c**) Chloroplasts expressing the GS fused with the N-terminal of SS4. Scale bars: 2 µm.
