# Supplementary material for: Coalescence and directed anisotropic growth of starch granule initials in subdomains of Arabidopsis thaliana chloroplasts
Source: Nat Commun. 2021 Nov 26;12:6944. doi: 10.1038/s41467-021-27151-5 (PMC8626487; doi:10.1038/s41467-021-27151-5)
Supplement: Supplementary file 3 — Description of Additional Supplementary Files [file 41467_2021_27151_MOESM3_ESM.pdf]

Description of Additional Supplementary Files

File Name: Supplementary Movie 1
Description: **SBF-SEM reveals that de-starched wild-type chloroplasts produce clusters of starch granule initials in the light.** Progression up through one SBF-SEM image stack, after which the direction of the movie reverses, leaving a semi-transparent rendering of the electron density (280 images, 50 nm Z-resolution). Starch granules (annotated in opaque violet) and the starch-containing pockets (annotated in transparent yellow) are embedded within the 3D-renderings. Note the high number of starch granules initials that can occupy a single pocket. For details of the imaged sample, see Fig. 1G and Fig. 2D.

File Name: Supplementary Movie 2
Description: **SBF-SEM reveals that clusters of starch granule initials appear to fuse as starch synthesis proceeds.** The movie is as described for Supplementary Movie S1 (345 images, 50 nm Z-resolution). Note the irregular morphology of larger granules within the pockets that appear to reflect the coalescence of starch granules initials. For details of the imaged sample, see Fig. 1H and Fig. 2D.

File Name: Supplementary Movie 3
Description: **SBF-SEM reveals normal lenticular starch granules at the end of the day.** The movie is as described for Supplementary Movie S1 (360 images, 50 nm Z-resolution). Note the small cluster size compared to earlier time points and the flat surfaces of abutting granules occupying the same pocket. Note also the variation in granule size and the comparatively large number of starchcontaining pockets. For details of the imaged sample, see Fig. 1I and Fig. 2D.

File Name: Supplementary Movie 4
Description: **Chloroplasts of wild-type mesophyll cells are de-starched after an extended night, as revealed by SBF-SEM.** Progression down through one SBF-SEM image stack (414 images, 50 nm Z-resolution) revealing multiple chloroplasts, all devoid of starch. Note the recurrent stromal spaces with a flocculate appearance that may represents sites for future starch granule initiation. For details of the imaged sample, see Fig. 1F.
